# Improving Electroactivity of N-Doped Graphene Derivatives with Electrical Induction Heating

**DOI:** 10.1021/acsaem.2c01184

**Published:** 2022-07-26

**Authors:** Miha Nosan, Luka Pavko, Matjaž Finšgar, Mitja Kolar, Boštjan Genorio

**Affiliations:** † Faculty of Chemistry and Chemical Technology, University of Ljubljana, Večna pot 113, Ljubljana SI-1000, Slovenia; ‡ National Institute of Chemistry, Hajdrihova 19, Ljubljana SI-1000, Slovenia; § Faculty of Chemistry and Chemical Engineering, University of Maribor, Smetanova ulica 17, Maribor SI-2000, Slovenia

**Keywords:** N-doped graphene nanoribbons, N-doped graphene, high specific surface area, induction heating, electrocatalysis, oxygen reduction reaction

## Abstract

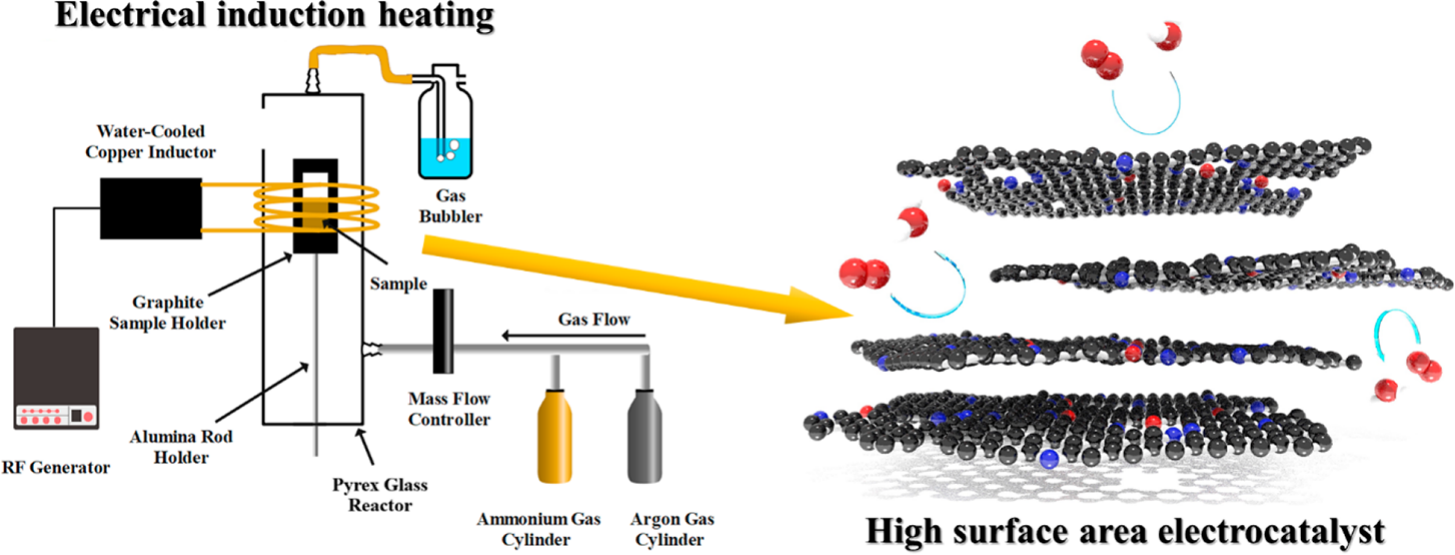

Graphene derivatives doped with nitrogen have already been identified as active non-noble metal materials for oxygen reduction reaction (ORR) in PEM and alkaline fuel cells. However, an efficient and scalable method to prepare active, stable, and high-surface-area non-noble metal catalysts remains a challenge. Therefore, an efficient, potentially scalable strategy to improve the specific surface area of N-doped graphene derivatives needs to be developed. Here, we report a novel, rapid, and scalable electrical induction heating method for the preparation of N-doped heat-treated graphene oxide derivatives (N-htGOD) with a high specific surface area. The application of the induction heating method has been shown to shorten the reaction time and improve the energy efficiency of the process. The materials synthesized by induction heating exhibited very high specific surface area and showed improved ORR activity compared to the conventional synthesis method. Moreover, we demonstrated that the temperature program of induction heating could fine-tune the concentration of nitrogen functionalities. In particular, the graphitic-N configuration increases with increasing final temperature, in parallel with the increasing ORR activity. The presented results will contribute to the understanding and development of nonmetal N-htGOD for energy storage and conversion applications.

## Introduction

The increasing demand for economic and environmentally friendly power generation has led to the development of energy storage and conversion devices such as batteries, supercapacitors, and fuel cells.^1−3^ The latter is gaining renewed interest as they are being considered to power fuel cell electric vehicles over long distances. The proton exchange membrane (PEM) fuel cell is the most mature among fuel cell technologies. However, active, selective, and stable electrode materials are required to achieve the desired performance of the PEM fuel cell. Nowadays, platinum-based electrode materials meet all these conditions, but platinum is a scarce, expensive metal and is susceptible to CO poisoning that needs to be replaced by a non-noble metal alternative. Therefore, an efficient non-noble metal material that lowers the cost and meets the energy performance of the PEM fuel cell is of great importance.^4^

Among non-noble metal materials, the class of heteroatom-doped nanocarbons, N-doped graphene derivatives, have great potential as electrocatalysts for the oxygen reduction reaction (ORR), which is considered the bottleneck of the PEM fuel cell. N-Doped graphene derivatives are environmentally friendly, low cost, tunable, and functionalizable, which makes them perfect candidates for electrode materials for low-temperature fuel cells.^4−7^

The methods used for the synthesis of N-doped graphene derivatives are diverse and can be classified as direct (chemical vapor deposition, segregation growth, solvothermal, and arc discharge) or post-treatment synthesis (thermal, laser, and hydrazine treatment) methods.^7^ Most of mentioned methods are time consuming and furnish low quantities of the final product, which is problematic in terms of future commercialization. However, if graphene oxide (GO) is used as a precursor for the synthesis of N-doped graphene derivatives, the process can be used with subsequent heat treatment for larger scale applications. However, depending on the GO synthesis conditions and GO heat treatment method, the complete restoration of the π- conjugated structure and exfoliation of N-doped heat-treated graphene oxide derivatives (N-htGOD) may not be fully achieved, resulting in a lower conductivity and lower specific surface area. Because high specific surface area and high electrical conductivity are of paramount importance for the electrochemical performance of N-htGOD as an electrode material, efficient methods need to be developed.^5,6,8^

To date, several different heating protocols have been reported for this process, e.g., Joule or resistive heating,^9−14^ ultrasonic heating,^15,16^ intense pulsed light,^17^ infrared,^18^ and microwave heating.^19,20^ In these protocols, the oxygen functionalities of GO are thermally decomposed, the nanostructure is exfoliated, and at the same time, the carbon nanostructure is N-doped, when a suitable nitrogen source is used.^7^ Depending on the post-treatment protocol and its parameters, the N-configurations can be doped into the structure in four different configurations, namely, pyridinic-N, pyrrolic-N, graphitic-N, and oxidized-N.^21−24^ Despite numerous research efforts to identify the active sites for ORR, there are conflicting claims about which N configuration is the most active. Some reports claim that ORR activity correlates with pyridinic-N,^25−30^ others claim that it is graphitic-N,^31^ whereas some claim that both functionalities contribute to ORR activity.^32^

Despite the influence of N configuration on ORR, many literature reports support the assumption that the superior ORR activity of N-htGOD is due to trace metal impurities.^33−40^

In our previous study,^41^ we showed that the aspect ratio of the graphene derivative, the nitrogen concentration, the specific surface area, and inherent metal impurities play an important role in enhancing the ORR activity. To further investigate the effects of surface area on N-htGOD materials, we developed a novel post-treatment protocol to generate higher-surface-area materials. A new one-pot nitrogen doping and heat-treatment method based on electrical induction heating was developed. In the present work, GO derivatives are heated with a heating rate up to 420 K/min in a graphite holder using a high-frequency electromagnetic field generated by an inductor. Is it to be noted that faster Joule heating procedures were also developed, utilizing the direct current discharge, with heating rates of 0.2–1 K/s, but they usually require complex instrumentation, are not scalable and the final temperature is difficult to control.^12−14^ All the synthesized N-htGOD, namely, N-doped heat-treated graphene oxide (N-htGO) and N-doped heat-treated graphene oxide nanoribbons (N-htGONRs), were subjected to a thorough morphological analysis and chemical and physical characterization as well as extensive electrochemical testing for ORR activity and stability in alkaline and acidic electrolytes.

## Experimental Section

### Materials

Graphite (Imerys, Timrex KS44), multiwalled carbon nanotubes (MWCNT) (NanoTechLabs M-grade MWCNT), KOH (Fluka, TraceSELECT, ≥ 99.995 wt%), HClO_4_ (Merck, Suprapure, 70 wt%), H_3_PO_4_ (Merck, ACS reagent, ≥ 85 wt% in H_2_O), H_2_SO_4_ (Merck, ACS reagent, 95.0–98.0 wt%), HCl (Merck, ACS reagent, 37 wt%), HF (Merck, SupraPur, 40 wt%), HNO_3_ (Merck, SupraPur, 65 wt%), KMnO_4_ (Merck, ACS reagent, ≥ 99.0 wt%), H_2_O_2_ (Merck, ACS reagent, 30 wt% in H_2_O) and (Honeywell Fluka, ISO, Ph.Eur grade, 65 wt%), 2-propanol (MiliporeSigma, HPLC grade, ≥ 99.7 wt%), Nafion (Merck, 5 wt%) perfluorinated resin solution were used as received. All dissolutions were performed with ultrapure water obtained from a Milli-Q system (Millipore) with a resistivity 18.2 MΩ cm.

### Preparation of Graphene Oxide (GO) and Graphene Oxide Nanoribbons (GONR)

For GO and GONR synthesis, we utilized of slightly modified and improved Hummers method,^42^ where a mixture of concentrated H_2_SO_4_/H_3_PO_4_ (vol. ratio = 900 mL:100 mL) was added to 20 g of graphite (Imerys, Timrex KS44) or 15 g of MWCNT (NanoTechLabs M-grade MWCNT) and then KMnO_4_ was added in aliquots (six aliquots of 20 g for graphite or eight aliquots of 15 g for MWCNT) with stirring, resulting in a mild exotherm to 35–40 °C. The reaction mixture was then stirred with a mechanical stirrer at room temperature in a 3 L beaker for 10 days. The reaction mixture was then poured onto ice (1500 mL), and 30 vol% H_2_O_2_ (approximately 15 mL) was added dropwise until the color changed from purple to yellow. Next, the mixture was transferred to 1 L plastic centrifuge bottles, diluted with ultrapure water, and centrifuged at 10 500 rpm for 30 min in a centrifuge (Sorvall LYNX 4000, Thermo Scientific). The supernatant was decanted, and the remaining solid was redispersed for 2 h in 5 vol% HCl ultrapure water solution. The last cleaning step comprised redispersing and soaking the GO/GONR suspension in ultrapure water until the next day. This was followed by centrifugation at 10 500 rpm for 1 h to discard the supernatant. This last cleaning step was repeated four times in total. After the final supernatant was discarded, GO/GONR was redispersed in ultrapure water. In the case of GO, the suspension was treated with a homogenizer (Ultraturrax T-25 basic, IKA) for 1 h at max rpm setting to exfoliate the product. In the case of GONR suspension, it was treated only with an ultrasonic bath (Iskra Sonis 4, Iskra) for 15 min. Finally, the GO/GONR were freeze-dried.

### Synthesis of N-htGO and N-htGONR by the Furnace Heating Method

Heat treatment was performed in an NH_3_ atmosphere at a constant flow rate of 30 mL/min in an aluminum oxide vessel in a quartz tube. The heat-treatment protocol consisted of three phases: (i) heating at 10 K/min from room temperature to the set point temperature (800 °C), (ii) maintaining the temperature at 800 °C for 10 min, and (iii) then cooling at average 5 K/min from the set point (800 °C) to room temperature.

### Synthesis of N-htGO and N-htGONR by the Induction Heating Method

Heat treatment was performed in an NH_3_ atmosphere at a constant flow rate of 30 mL/min in a graphite (99.9 wt%) crucible inside a Pyrex glass reactor using the induction heat-treatment protocol: (i) heating from room temperature to 800 or 1200 °C (heating rate of 420 K/min), (ii) holding at 800 or 1200 °C for 2 min, and (iii) cooling from 800 or 1200 °C to room temperature (average cooling rate of 50 K/min). The heat was generated by an HTG-2400 inductor (Induktio) operating at a frequency of 250 Hz and an output power of 1700 W.

### Preparation of the Thin Film Working Electrode (WE)

The catalyst ink was prepared by mixing the sample (N-htGO or N-htGONR), ultrapure water, 2-propanol (IPA), and Nafion (5 wt% water resin) in a ratio of 4.5 mg:0.8 mL:0.3 mL:30.0 μL, respectively. The mixture was then homogeneously dispersed with a horn sonicator (37.5 W). Finally, an aliquot of 25 μL was applied to a glassy carbon disk electrode (diameter = 5.5 mm) on an inverse rotating ring disk electrode (RRDE), equipped with a gold ring electrode at a rotation of 300 rpm at room temperature for 45 min.

### Electrochemical Characterization

#### ORR Activity

Cyclic voltammetry (CV) measurements were performed with PGSTAT30 (Autolab) potentiostat/galvanostat with the three-electrode system using a reference electrode (Basi Research Products RE 5B) Ag/AgCl in 3 M NaCl, mixed metal oxide (IrO_2_ and TiO_2_ on Ti support, Specialist Casting) as the counter electrode, and rotating ring disk electrode (RRDE) (Pine Research) with glassy carbon disk and gold ring electrode as the working electrode. RRDE experiments were performed at 1600 rpm on a WaveVortex 10 (Pine Research) rotation control unit. For all electrochemical experiments, 0.1 M KOH or 0.1 M HClO_4_ aqueous electrolytes saturated with Ar or O_2_ were used at scan rate of 20 mV/s. Electrolyte resistance was determined by the positive feedback method before each measurement and total solution resistance compensation (90%) was performed. All potentials in this work refer to the RHE (V vs. RHE). Before each measurement, the potential of the Ag/AgCl (3 M NaCl) RE was checked by measuring the opening circuit potential of the reference electrode with respect to a platinum wire, both immersed in an H_2_-saturated solution (0.1 M HClO_4_ or 0.1 M KOH, respectively). We compared the ORR activity of the N-htGOD by the onset potential (*E*_onset_), determined by the tangential method.^43^

#### Electrochemical Stability

Chronoamperometric stability measurements were performed with RRDE in the range of kinetic-diffusion current at 0.70 V vs. RHE and 0.30 V vs. RHE in 0.1 M KOH and 0.1 HClO_4_, respectively, at 1600 rpm for a duration of 2 h.

#### Number of Transferred Electrons

The number of transferred electrons per reduced oxygen molecule on the RRDE (*n*) was determined using the following equation:
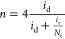
Where *i*_d_ is the disk current, *i*_r_ is the ring current, and *N*_c_ (0.38) is the collection efficiency. *N*_c_ was determined in 10 mM Fe(CN)_6_^3–^/Fe(CN)_6_^4–^ at 1600 rpm in 0.1 M HClO_4_ solution.

### Scanning Electron Microscopy (SEM)

Microstructure characterization of N-htGOD was performed using a SEM (Zeiss ULTRA plus). SEM images were acquired at 2 kV with secondary electron detector (SE2) at a working distance of 5.5 mm.

### Brunauer–Emmett–Teller Analysis (BET)

Prior to BET analysis, N-htGOD were degassed under a vacuum (10 μmHg) at 120 °C for 2 h. Subsequently, the BET specific surface area was determine on the ASAP 2020 Micromeritics instrument with N_2_ gas adsorption at 77 K.

### X-ray Photoelectron Spectroscopy (XPS) Measurements

XPS measurements were performed using a Supra plus spectrometer (Kratos, Manchester, UK) equipped with a hemispherical analyzer and a monochromatic Al K_α_ X-ray source. Survey spectra were measured using a pass energy of 160 eV with a step of 1 eV/s. High-resolution spectra were measured using a pass energy of 20 eV at a step of 0.1 eV/s. The data were acquired using the *ESCApe 1.4* software. Fitting of high-resolution XPS spectra was performed using CasaXPS software, with Shirley background subtraction using 30–70% Gaussian–Lorentzian peak shapes, except for the oxidized-N configuration where additional asymmetric peak tailing was used. The oxidized-N configuration was fitted with an asymmetric peak shape, using 1.2–1.6 eV constrain for full width at the half-maximum . The binding energy was corrected using the C–C/C–H peak in the C *1s* spectra at 284.5 eV.

### Raman Spectroscopy

Raman spectroscopy was performed using the Raman/AFM WITec Alpha 300 RAS with a 532 nm laser light with a power of 15 mW and 30 s integration time. Peak fitting of Raman spectra was performed with the Lorentzian peak shape, baseline constrain at 0 intensity and peak at center constrains shown in Table S1.^44^

### Inductively Coupled Plasma Mass Spectrometry (ICP-MS)

Trace element analysis of electrocatalysts was determined using an ICP-MS Agilent Technologies 7900CE, with a micro nebulizer, quartz spray chamber, and quadrupole mass analyzer in the flow of high-purity argon (5.0) gas at a flow rate of 15 L/min. The data were processed and analyzed using *MassHunter 4.4* software.

#### Sample Preparation–Acid-Assisted Microwave Digestion

Approximately 50 mg of the catalyst powder was placed into a 50 mL autoclave and 4 mL of H_2_SO_4_ (96 wt%, Fluka – Honeywell, puriss), 3 mL HNO_3_ (65 wt%, Merck, suprapur), 2 mL of HClO_4_ (70 wt%, Merck, suprapur), and 1 mL HF (40 wt%, Merck, suprapur) were added. The mixture was heated according to the temperature program: to 150 °C for 15 min, 220 for 20 min, 230 for 15 min, and then cooled in a microwave digestion system (Milestone Ethos UP). Next, the mixture was transferred to a 50 mL flask and diluted to the mark with ultrapure water. Finally, the as-prepared solution was filtrated through a 0.45 μm filter, diluted by a factor of 100 with 1% HNO_3_ (65%, Merck, suprapur) solution, and injected into the ICP-MS instrument.

### Analysis of Bulk Electrical Conductivity Using the Four-Point Probe Method

We performed four-point probe ex situ conductivity (Ossila) measurements^45^ on powder N-htGOD compressed (using a manual FTIR KBr hydraulic pellet press) at 8 tons.

## Results and Discussion

### Synthesis and Characterization (Morphological, Chemical, and Spectroscopical)

**Figure 1:**
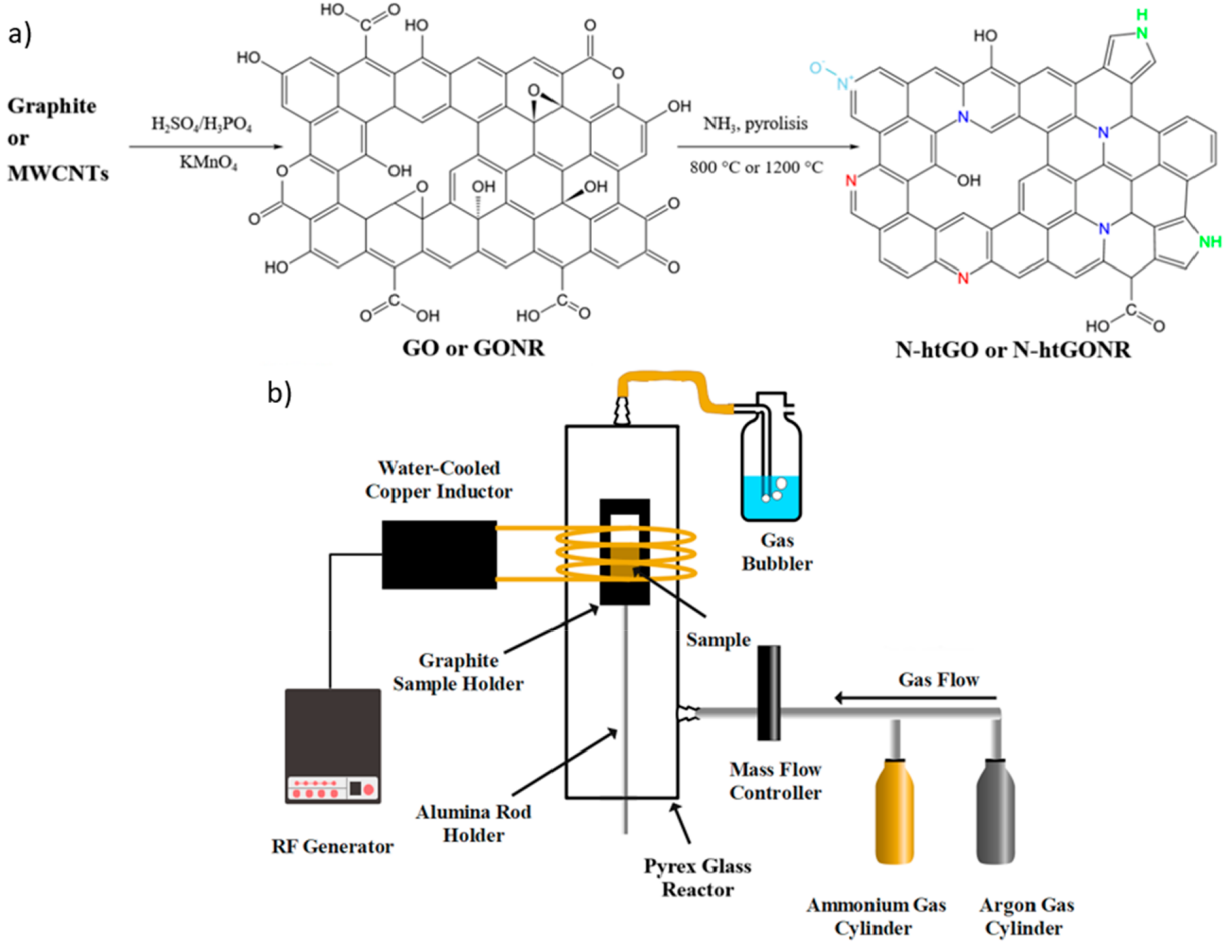
Schemes of (a) synthesis procedure and (b) induction heating setup.

Different aspect ratio materials: Quasi-1D N-htGONR and 2D N-htGO were prepared by a two-step, top-down post-treatment synthesis approach Figure 1a, using two different heating methods. In the first synthesis step, the starting materials graphite or multiwalled carbon nanotubes (MWCNT) were oxidized by an improved Hummers method and freeze-dried. Freeze-drying is an important step that enables an improved specific surface area of graphene oxide (GO) and graphene oxide nanoribbons (GONR).^46^ In the next step, the prepared GO and GONR were heat treated up to 800 or 1200 °C in a NH_3_ atmosphere using two different heating methods, i.e., conventional furnace heating (Figure S1) and novel electrical induction heating (Figure 1b).

The first difference between the heat treatment protocols is in the crucible used. Furnace heating uses nonconductive alumina, whereas induction heating uses a graphite crucible as the reactor. The high-purity graphite reactor used in induction heating is essential because it does not contaminate the sample and is an extremely good heat conductor. Further, the magnetic current generated by the induction coil induces electric currents in the graphite material. Because of the internal resistance of the graphite, the graphite reactor immediately generates heat that is transferred to GO or the GONR sample. The heating temperature and heating rate can be adjusted with the current flowing through the induction coil. The higher the power input, the more heat is generated and transferred to the sample.^19^ Because of the high power of the induction generator, we achieved rapid heating rates as high as 420 K/min that far surpass the conventional furnace heating of 50 K/min. In addition, the type of induction heating setup used in the heating and cooling steps significantly shortens the synthesis time. This enables a shorter reaction time, lower cost, consumption of water and carrier gas and better energy efficiency of the process compared to conventional furnace heating. The entire induction heat treatment cycle took only 15 min, whereas the conventional furnace required 240 min. Induction heating thus meets the guidelines for efficient industrial processes.

**Figure 2:**
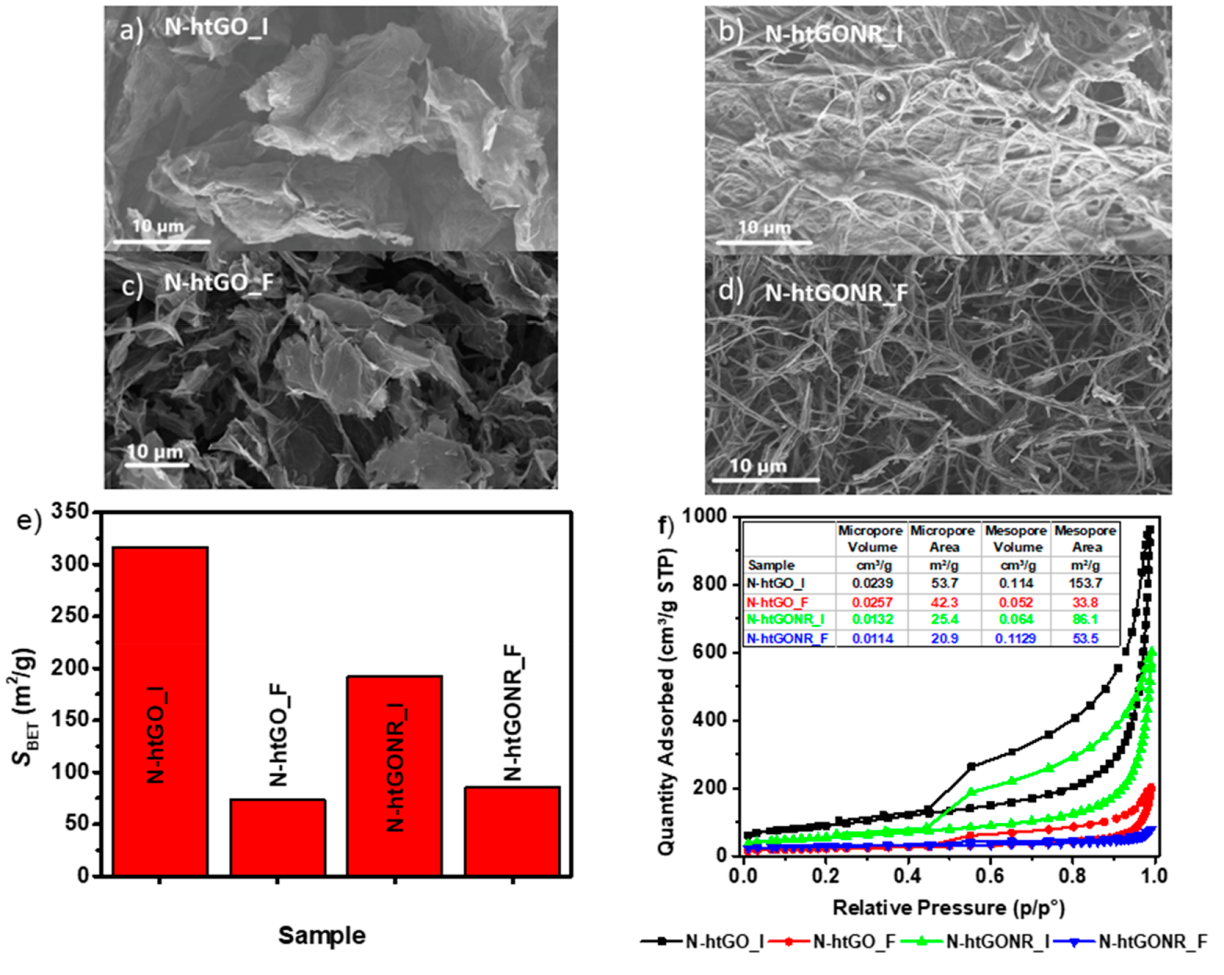
SEM images of (a) N-htGO_I, (b) N-htGONR_I, (c) N-htGO_F, (d) N-htGONR _F, (e) specific BET surface area, and (f) BET isotherms for nitrogen adsorption capacity of N-htGO_I, N-htGONR_I, N-htGO_F, and N-htGONR_F with micropore and mesopore volume and area.

The N-htGO and N-htGONR prepared by two heating procedures were further characterized and compared morphologically, chemically, and electrochemically. N-htGO_F/N-htGONR_F indicates N-htGO and N-htGONR prepared with the furnace, and N-htGO_I/N-htGONR_I for N-htGO and N-htGONR that were prepared with the induction heating setup. The morphology of the two N-htGO is shown in Figure 2a, c and two N-htGONR in Figure 2b, d. Different heating procedures showed a similar morphology for N-htGO—both materials had wrinkled flake structures with an average lateral size of 20 μm. Similarly, N-htGONR materials in Figures 2b, d. had a net covered sheetlike structure. The most significant differences in morphology were present by the nitrogen adsorption technique using the BET method. As can be seen in Figure 2e, all N-htGOD prepared by induction heating showed evidently higher surface area compared to furnace heating method. The N-htGOD with the highest surface area of 317 m^2^/g was N-htGO_I, followed at 192 m^2^/g by N-htGONR_I, N-htGONR_F at 85 m^2^/g, and N-htGO_F at 74 m^2^/g. Inductively heated specimens were expected to have a larger surface area because rapid heating resulted in better thermal exfoliation. A higher heating rate resulted in more efficient thermal expansion of the graphene oxide material and thus graphene derivatives with a large specific surface area.^47^ The better exfoliation of the induction heat-treated N-htGOD was also confirmed by Raman spectra, especially by comparing the intensities between 2D and G peaks. A higher 2D/G ratio indicates better exfoliation. As shown in Raman spectra (Figure S2) the intensity ratio of the 2D/G peak is higher for N-htGO_I (0.16) and N-htGONR_I (0.12) relative to N-htGO_F (0.08) and N-htGONR_F (0.09). One can also speculate from the shape of the gas adsorption isotherms, pore volume and pore area shown in Figure 2f that the high specific surface area is a result of better exfoliation and not a different porosity type.^48^

**Figure 3:**
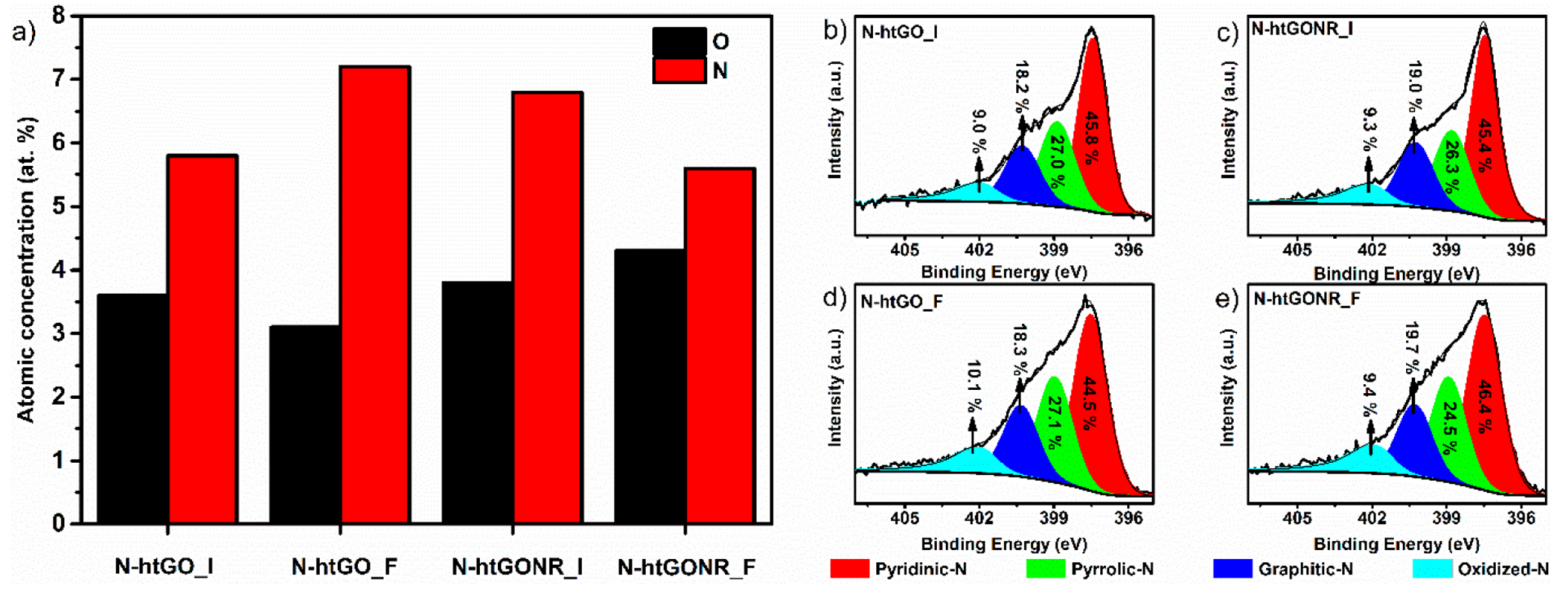
Schematic representation of (a) surface atomic concentration of O and N in graphene derivatives, and N *1s* fitted XPS spectra of (b) N-htGO_I, (c) N-htGONR_I, (d) N-htGO_F, and (e) N-htGONR_F. All of the N-functionalities are given relative to the total N at% concentration in the N-htGOD.

Because the surface chemical composition of these materials is vital for electrocatalysis applications, XPS and Raman spectroscopy measurements were performed to determine the elemental composition, nitrogen functionalities, and defectiveness of graphene derivatives. The Raman spectra and XPS survey are shown in Figures S2 and S3, respectively. The atomic concentrations of O and N for different N-htGOD were determined using high-resolution XPS spectra and are presented in Figure 3a. Different heating methods result in O and N surface atomic concentrations in the range of 3.1–4.3 at% and 5.6–7.2 at%, respectively. The latter indicates that different heating protocols do not significantly affect the O and N atomic surface concentrations. Next, the N *1s* spectra fitting was performed to determine the nitrogen functionalities in different N-htGOD. The results presented in Figure 3b–e show that the N-htGOD prepared by different methods have similar concentrations of N-containing species. The difference between the N-configurations was less than 1.3%. The percent for the N-functionalities are given relative to the total N at concentration in the sample. On the other hand Raman spectroscopy does not show significant differences between the N-htGOD structures.

Next, the ICP-MS analysis of N-htGOD was performed to determine the composition of trace metal impurities. The impurity concentrations are given in Table S2. Main impurities detected in ppm were K, Mn, and Fe. The Mn and K impurities are introduced during the oxidation step of graphite or MWCNT with KMnO_4_, and the Fe is an inherent impurity present in the starting graphite material or MWCNT. As we have shown in our previous work,^41^ the presence of Fe affects the ORR activity of the graphene derivatives in KOH, and thus, starting materials with low concentrations of inherent Fe were used in the present study.

### Electrochemical Characterization (ORR Activity, Selectivity, and Stability)

**Figure 4:**
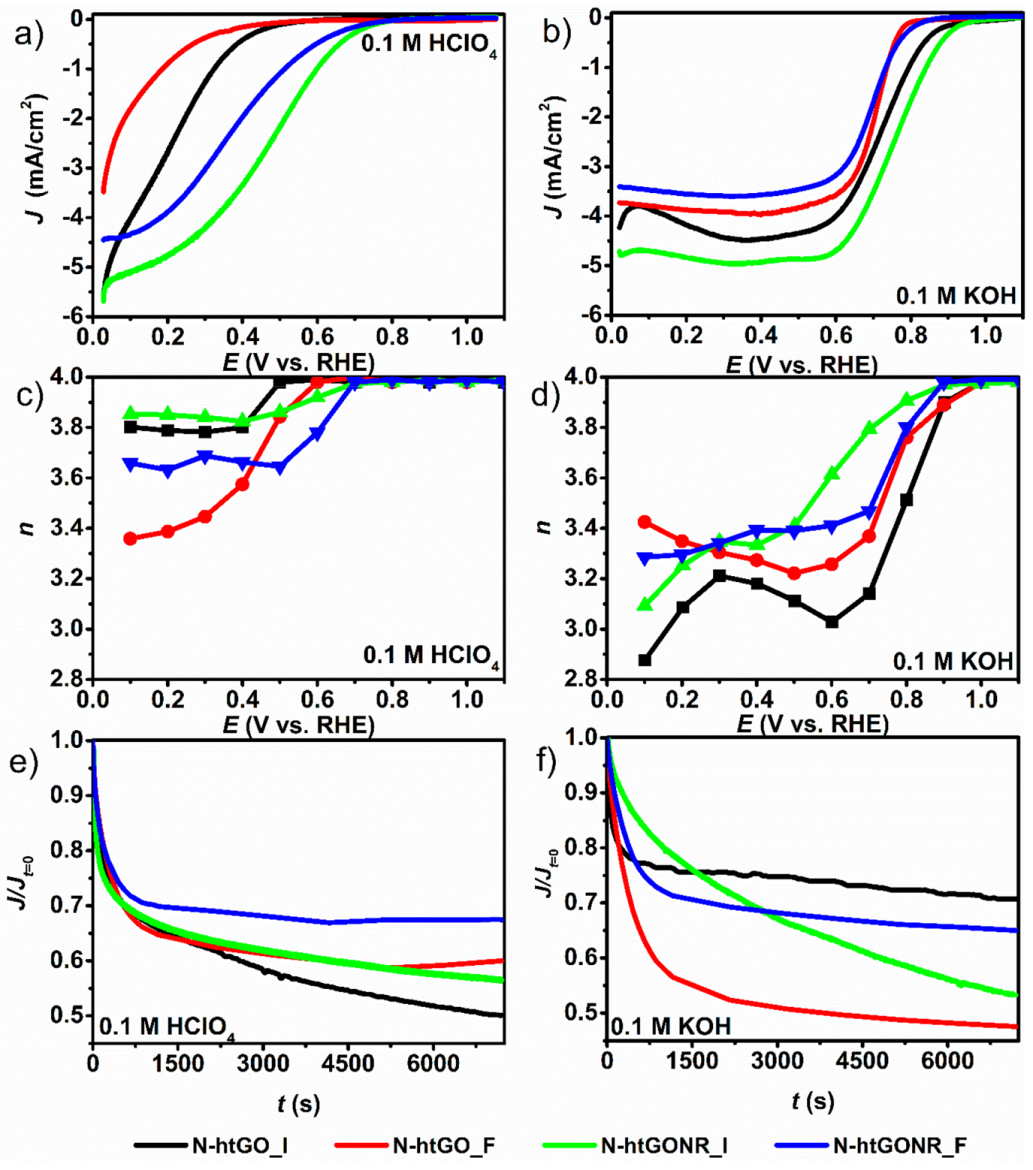
Electrochemical measurements of N-htGO_I (black curves), N-htGO_F (red curves), N-htGONR_I (green curves), and N-htGONR_F (blue curves) materials; ORR activity in (a) 0.1 M HClO_4_ and (b) 0.1 M KOH, selectivity in (c) 0.1 M HClO_4_ and (d) 0.1 M KOH and electrochemical stability in (e) 0.1 M HClO_4_ and (f) 0.1 M KOH at 0.3 and 0.7 V vs. RHE, respectively. Electrochemical measurements were performed in an O_2_ saturated solution at a scan rate of 20 mV at rotation rate of 1600 rpm.

The above-mentioned chemical composition showed that the N-htGOD prepared by two different heating procedures are very similar. On the other hand, the morphological data clearly show that the materials prepared by the induction heating method are more exfoliated and therefore have a considerably larger specific surface area. The latter should lead to a larger number of accessible active sites. On this basis, the materials were tested for ORR activity, selectivity, and stability in acidic 0.1 M HClO_4_ and alkaline 0.1 M KOH electrolytes. Figure 4a, b shows the ORR activity in 0.1 M HClO_4_ and 0.1 M KOH, respectively. ORR cyclic voltammograms were compared, in particular the *E*_onset_ for reduction.

The *E*_onset_ values of the N-htGOD prepared by induction heating are considerably higher in both electrolytes (Table 1). In 0.1 M HClO_4_, the *E*_onset_ values were: 0.324 V (N-htGO_I), 0.186 V (N-htGO_F), 0.512 V (N-htGONR_I) and 0.477 V (N-htGONR_F) vs. RHE. A similar trend with higher *E*_onset_ was determined in 0.1 M KOH. The *E*_onset_ values were 0.795 V (N-htGO_I), 0.726 V (N-htGO_F), 0.841 V (N-htGONR_I), and 0.737 V (N-htGONR_F) vs. RHE. Moreover, as shown previously,^41^ the trend of increasing aspect ratio of graphene derivative corresponds with increasing electrocatalytic activity for ORR. In this respect, quasi-1D N-htGONR always performed better than 2D N-htGO.

**Table 1:** Elemental Concentration (at%) of C, O, and N Determined by XPS, Specific BET Surface Area, and *E*_onset_ in 0.1 M KOH and HClO_4_ for N-htGO_I, N-htGO_F, N-htGONR_I, and N-htGONR_F.

N-htGOD	C (at%)	O (at%)	N (at%)	pyridinic-N (at%)	*S*_BET_ (m^2^/g)	*E*_onset_ (KOH) (V vs. RHE)	*E*_onset_ (HClO_4_) (V vs. RHE)
N-htGO_I	90.6	3.6	5.8	2.66	316.8	0.795	0.324
N-htGO_F	89.7	3.1	7.2	3.20	74.1	0.726	0.186
N-htGONR_I	89.3	3.8	6.7	3.04	192.0	0.841	0.512
N-htGONR_F	90.1	4.3	5.6	2.60	85.4	0.737	0.477

Next, the selectivity for ORR was measured and compared using RRDE. ORR occurs mainly by two pathways: 2*e*^–^ reduction to peroxide or 4*e*^–^ direct reduction to water. For fuel cell applications, 4*e*^–^ reduction is desirable because of better energy efficiency.^49^ The N-htGOD prepared by induction heating in 0.1 M HClO_4_ showed better selectivity for the direct 4*e*^–^ pathway than that prepared by furnace heating (Figure 4c). In alkaline 0.1 M KOH electrolyte, all the materials show worse selectivity and no apparent trend (Figure 4d) compared to 0.1 M HClO_4_. On the basis of these selectivity data measured in the different electrolytes, we can conclude that all induction heated materials exhibit higher selectivity for 4*e*^–^ pathway in acidic 0.1 M HClO_4_ electrolyte compared to alkaline 0.1 M KOH electrolyte.

Finally, the electrochemical stability of the N-htGOD was evaluated. The results revealed that furnace prepared N-htGO_F and N-htGONR_F show better stability than induction heated high specific surface area N-htGO_I and N-htGONR_I materials (Figure 4e, f). This is in line with the general observation where more active catalysts show poorer stability.^50^ However, we attributed the poorer stability of induction-heated N-htGOD to a larger specific surface area and thus greater exposure of active sites to the electrochemical reaction and electrolyte, followed by a faster degradation that may be a result of the oxidation of the carbon backbone, because the carbon oxidation is thermodynamically possible at potentials above 0.2 V vs. RHE.^51^

In conclusion, the N-htGOD morphology, i.e., a larger surface area, increased ORR activity in both alkaline and acidic electrolytes. A higher aspect ratio had the same effect on ORR, with quasi-1D materials performing better than 2D materials. All of the materials showed excellent selectivity for 4*e*^–^ transfer with improved selectivity in acidic electrolytes compared to alkaline electrolytes. And finally, the electrochemical stability of N-htGOD was negatively affected by a higher specific surface area.

### Influence of N-Configuration on the ORR Activity

**Figure 5:**
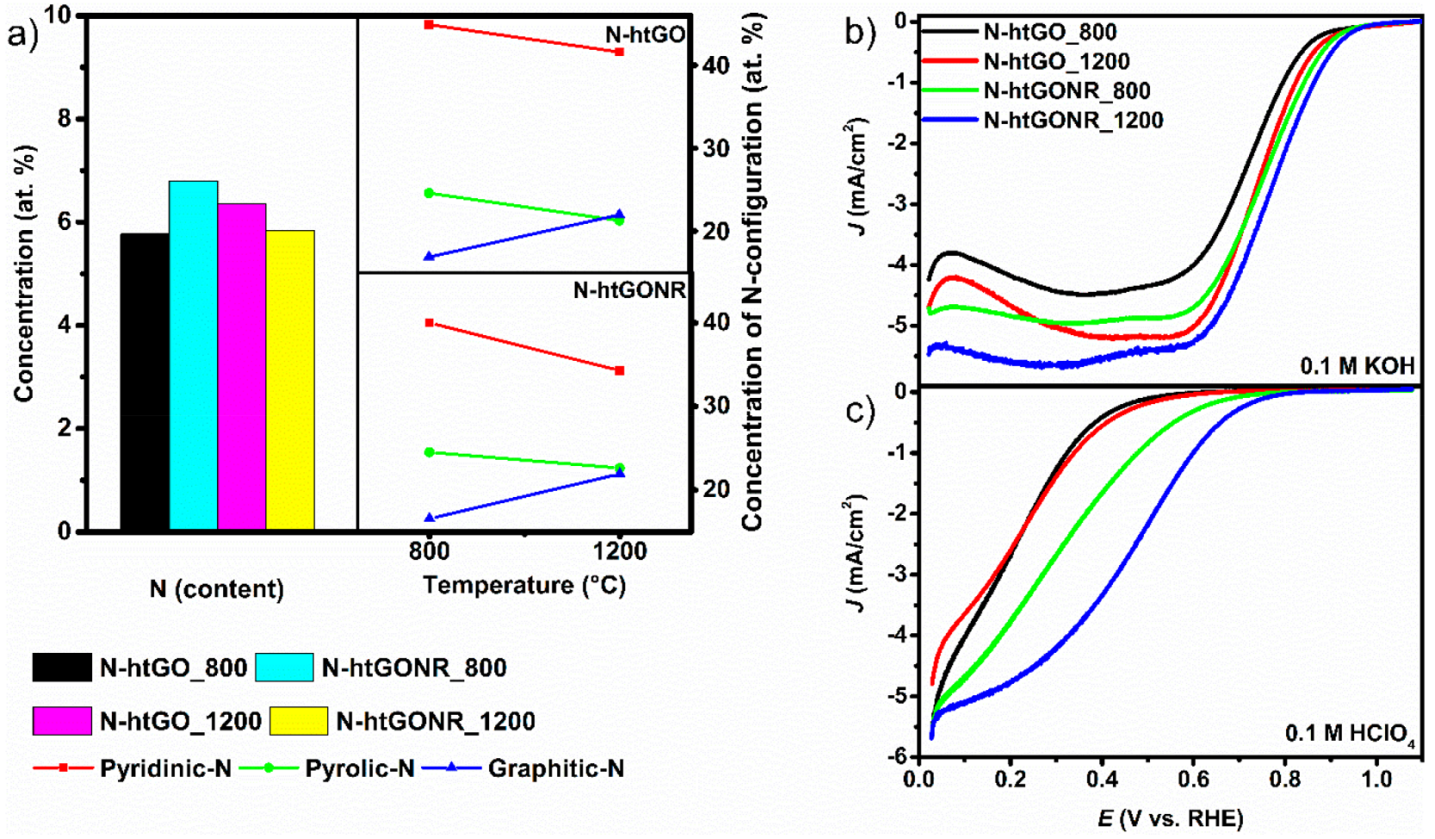
(a) Representation of N concentration and N-configuration distribution in N-htGO and N-htGONR heat treated at 800 and 1200 °C, respectively, and ORR activity performance in (b) 0.1 M KOH and (c) 0.1 M HClO_4_. Electrochemical measurements were performed in an O_2_-saturated solution at a scan rate of 20 mV at rotation rate of 1600 rpm.

In the systematic study, we also investigated induction-heated N-htGONR and N-htGO materials prepared by the same heating rate but at different final temperatures. One set of N-htGOD was prepared at the final temperature of 800 °C (N-htGO_800 and N-htGONR_800) and the other set at 1200 °C (N-htGO_1200 and N-htGONR_1200). A SEM morphological comparison of both sets (Figure S4) shows no distinctive morphological difference. However, chemical, and electrochemical characterization interestingly show a trend (Figure 5).

The graphene derivatives N-htGO_1200 and N-htGONR_1200 showed significantly higher ORR activity in alkaline and acidic electrolyte compared to N-htGO_800 and N-htGONR_800. Figure 5a shows that the N-configuration determined by N *1s* XPS spectra (Figure S5) varies with the final temperature, whereas there is no significant difference in overall nitrogen concentrations. The materials synthesized at 1200 °C showed a lower concentration of pyridine-N and pyrrole-N configuration and a higher concentration of graphitic-N. On the other hand, materials synthesized at 800 °C showed a higher concentration of pyridine-N and pyrrole-N configuration and a lower concentration of graphitic-N. When N-configuration was correlated to the electrochemical results, it was evident that nitrogen configuration strongly impacted the electrochemical activity (Figures 5b, c). We hypothesize that the graphitic-N configuration is responsible for the improved ORR activity. Our findings contrast with the majority of literature reports, where pyridinic N is believed to have the highest impact on ORR activity. However, there are several reports that are in contradiction, and the authors have come to a different conclusions.^31,32^

In view of the present results, we believe the electrocatalytic performance is not related to only one parameter but is a sum of several parameters. We have shown in Figure 5 that N-htGOD prepared at 1200 °C and higher graphitic-N concentration had better ORR activity, but on the other hand, the N-htGOD have much lower O concentrations (shown in XPS survey spectra in Figure S6 and Table S3), which is an indicator of restored graphene structure thus improving the conductivity of the prepared electrocatalyst. To quantify the electrical conductivity of N-htGO_800, N-htGO_1200, and N-htGONR_800 and N-htGONR_1200, we performed ex situ four-point probe measurements. The results showed improved electrical conductivity for the materials heat treated at 1200 °C (Table S4). Improved electrical conductivity is also an important property for effective electrocatalysis. To fully understand the main factor for the improved activity of the presented electrocatalyst, we need to perform additional systematic studies on defined surfaces, which would give a better insight into the electrocatalytic properties. Nonetheless, the present results show that the specific surface area, the N-configuration, and the aspect ratio should be considered when designing a non-noble metal electrocatalyst for ORR.

Finally, we also compared the ORR activity of N-htGOD with the *E*_onset_ potential (Tables S5 and S6). The best performing material N-htGONR_1200 showed high *E*_onset_ in 0.1 M KOH and 0.1 M HClO_4_. This was attributed to the synergistic catalytic effect of the specific surface area, the N-configuration, and the aspect ratio

## Conclusion

We have synthesized and characterized quasi 1D N-doped heat-treated graphene oxide derivatives (N-htGONR) and 2D analogs (N-htGO) using induction and furnace heating methods at different temperatures. In addition, to rule out the influence of trace metal impurities on ORR performance, we treated the same materials with different heat-treatment protocols.

The application of this method reduces the need for large furnaces, shortens the reaction time, and improves the energy efficiency of the heat treatment process. The main advantage of the induction heating method compared to the furnace method is the faster heating rate, which produces graphene derivatives with a larger surface area that exhibits improved ORR activity in alkaline and acid electrolytes. We have confirmed that a higher aspect ratio improves ORR activity, with quasi-1D N-htGONR exhibiting higher activity than 2D N-htGO. In addition, the RRDE experiments showed that the inductively nitrogen-doped heated-treated graphene oxide derivatives (N-htGOD) exhibited better selectivity for the 4*e*^–^ reduction in acidic electrolytes than the N-htGOD prepared by the conventional furnace method. However, the stability of the inductively heated N-htGOD decreased with a larger surface area. When comparing the ORR performance between N-htGOD heat treated at different final temperatures, it was also found that the increase in the concentration of graphitic-N could be responsible for the increased ORR activity. In summary, the present results indicate that the specific surface area, the N-configuration, and the aspect ratio should be considered in the development of new non-noble metal electrocatalysts for ORR. Moreover, the results indicate that the electrical induction heating protocol is energy and time efficient and can be scaled up industrially. This is a step toward a low-cost active non-noble metal catalyst for the ORR reaction in PEM and alkaline fuel cells.

## Supporting Information Available

Additional SEM, Raman, XPS Figures, ICP-MS, and electrochemical results (PDF)
