# Supplementary material for: Improving Electroactivity of N-Doped Graphene Derivatives with Electrical Induction Heating
Source: ACS Appl Energy Mater. 2022 Jul 26;5(8):9571–80. doi: 10.1021/acsaem.2c01184 (PMC9400296; doi:10.1021/acsaem.2c01184)
Supplement: Supplementary file 1 — ae2c01184_si_001.pdf [file ae2c01184_si_001.pdf]

Improving Electroactivity of N-Doped Graphene Derivatives with Electrical Induction Heating

*Miha Nosan,[1] Luka Pavko,[1, 2] Matjaž Finšgar,[3] Mitja Kolar,[1] and Boštjan Genorio[1]**

[1]Faculty of Chemistry and Chemical Technology, University of Ljubljana, Večna pot 113, SI-1000 Ljubljana, Slovenia

[2]National Institute of Chemistry, Hajdrihova 19, SI-1000, Ljubljana, Slovenia

[3]Faculty of Chemistry and Chemical Engineering, University of Maribor, Smetanova ulica 17, SI-2000 Maribor, Slovenia

*E-mail: bostjan.genorio@fkkt.uni-lj.si

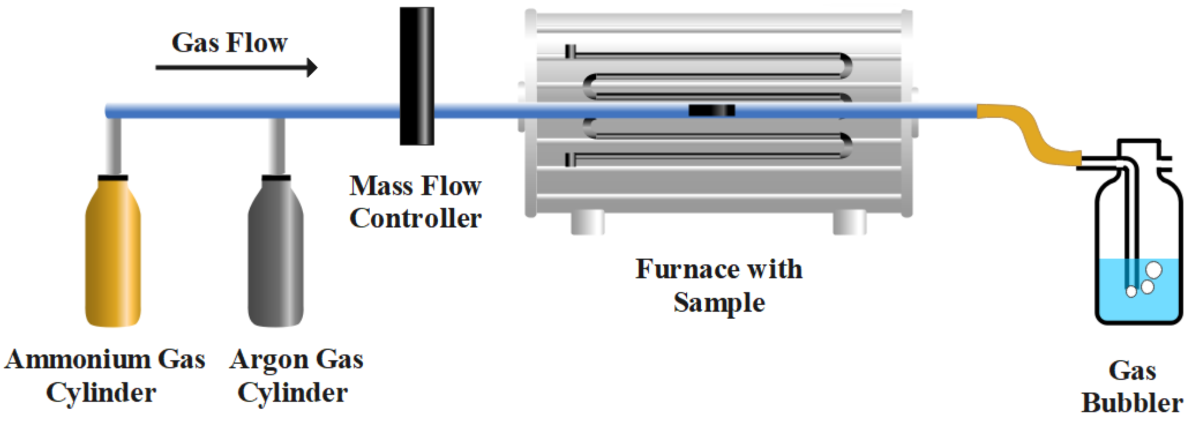

Figure S1: Scheme of furnace heating setup.

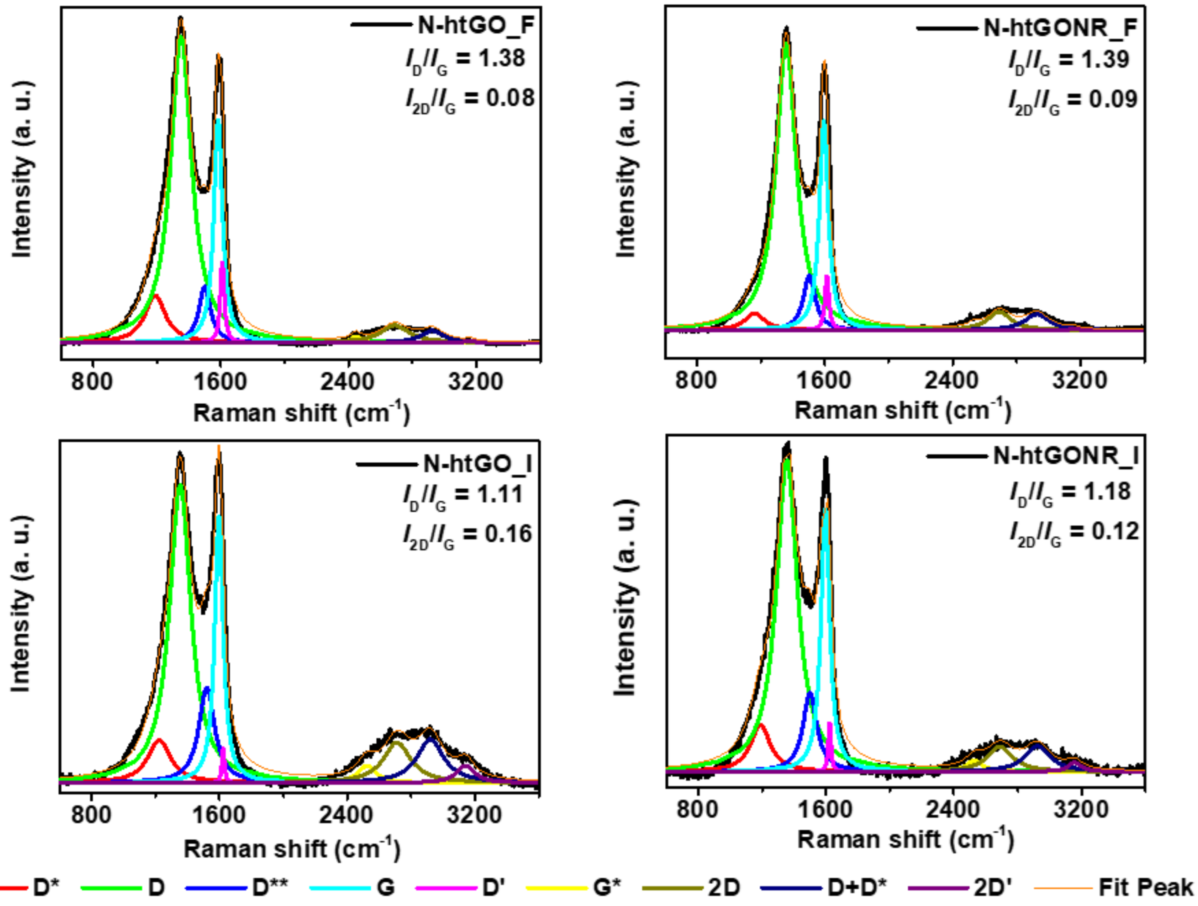

Figure S2: Peak fitted Raman spectra of a) N-htGO_F, b) N-htGONR_F, c) N-htGO_I and d) N-htGONR_I materials.

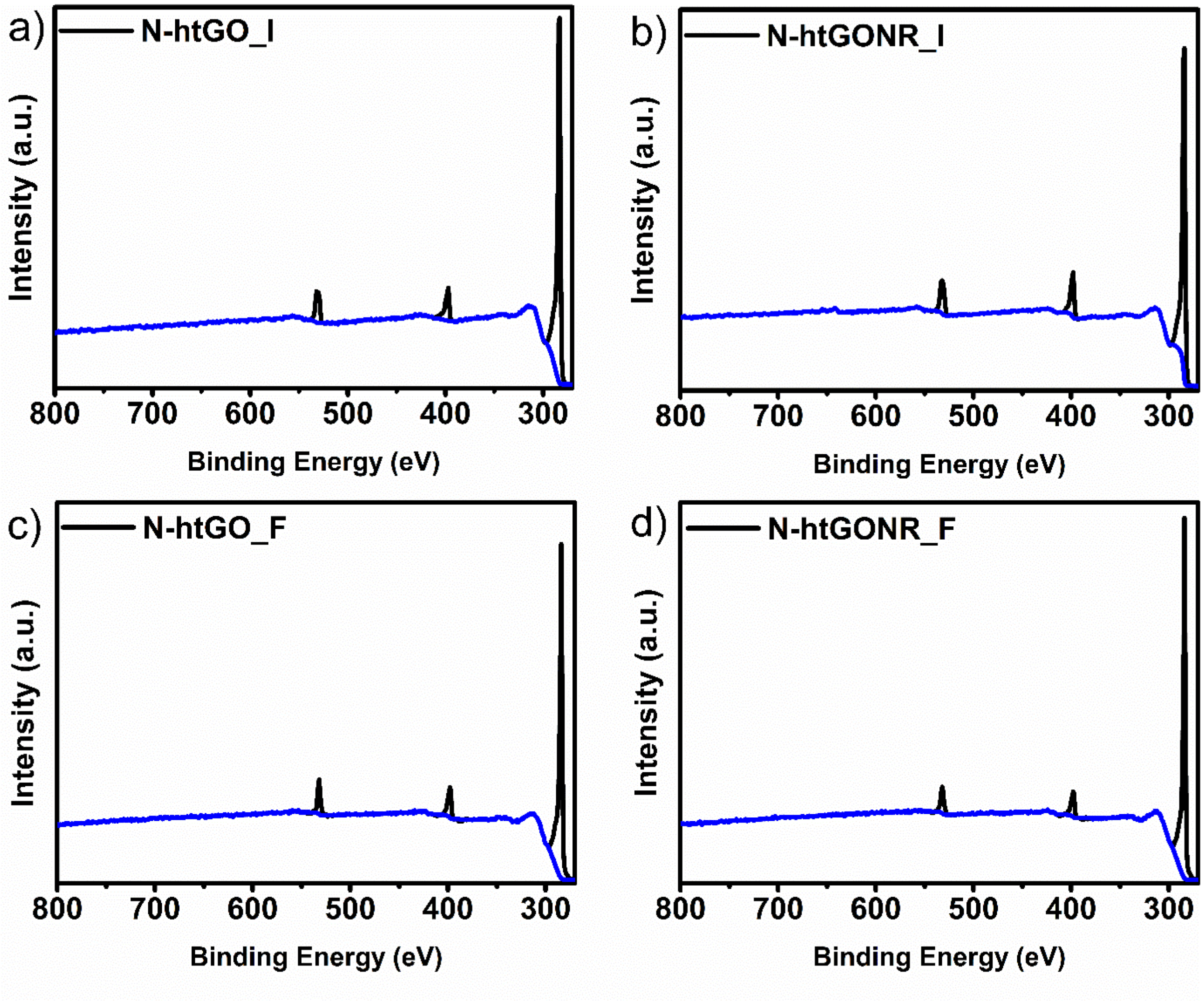

Figure S*3*: XPS survey spectra of a) N-htGO_I, b) N-htGONR_I, c) N-htGO_F and d) N-htGONR_F materials.

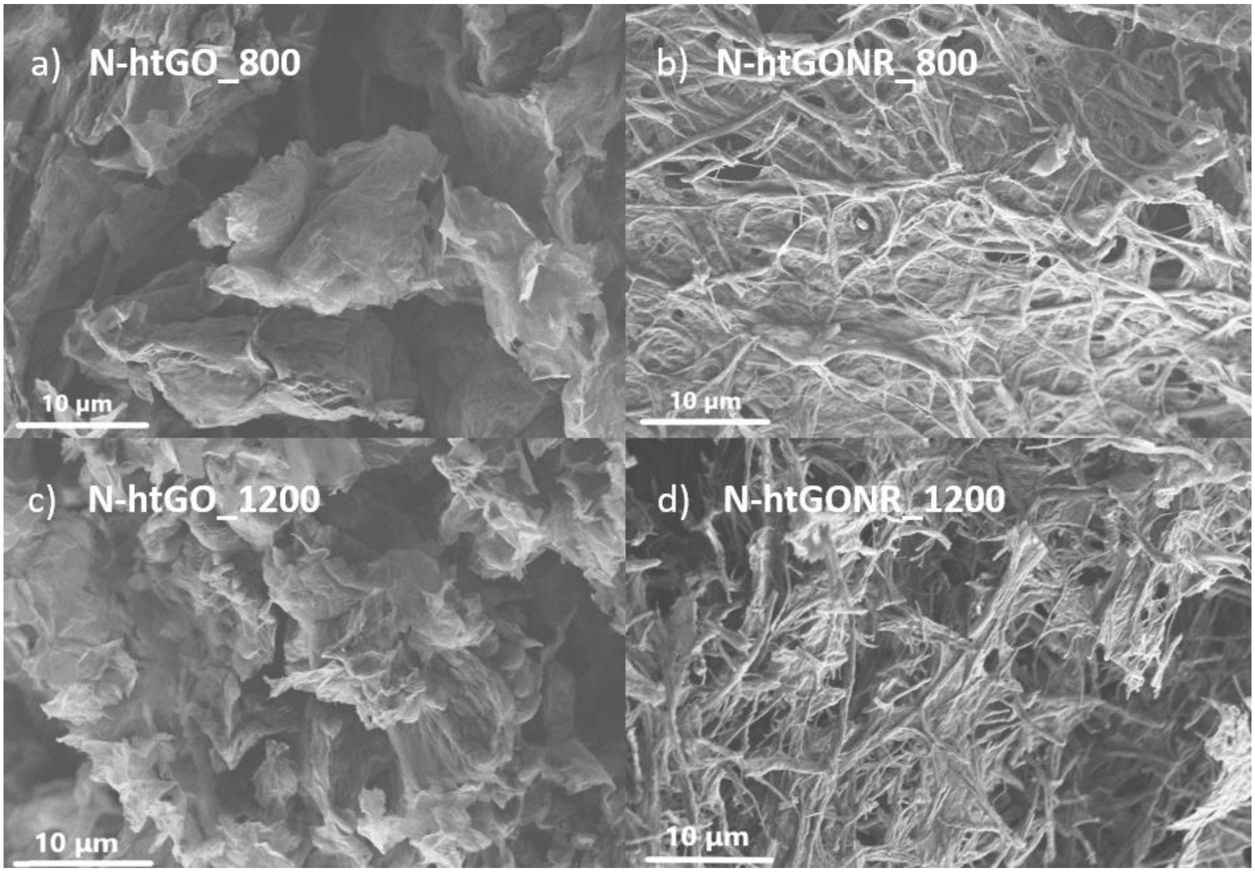

Figure S*4*: SEM images of a) N-htGO_800, b) N-htGONR_800, c) N-htGO_1200, d) N-htGONR_1200 materials prepared by induction heating protocol at temperature 800 °C or 1200 °C.

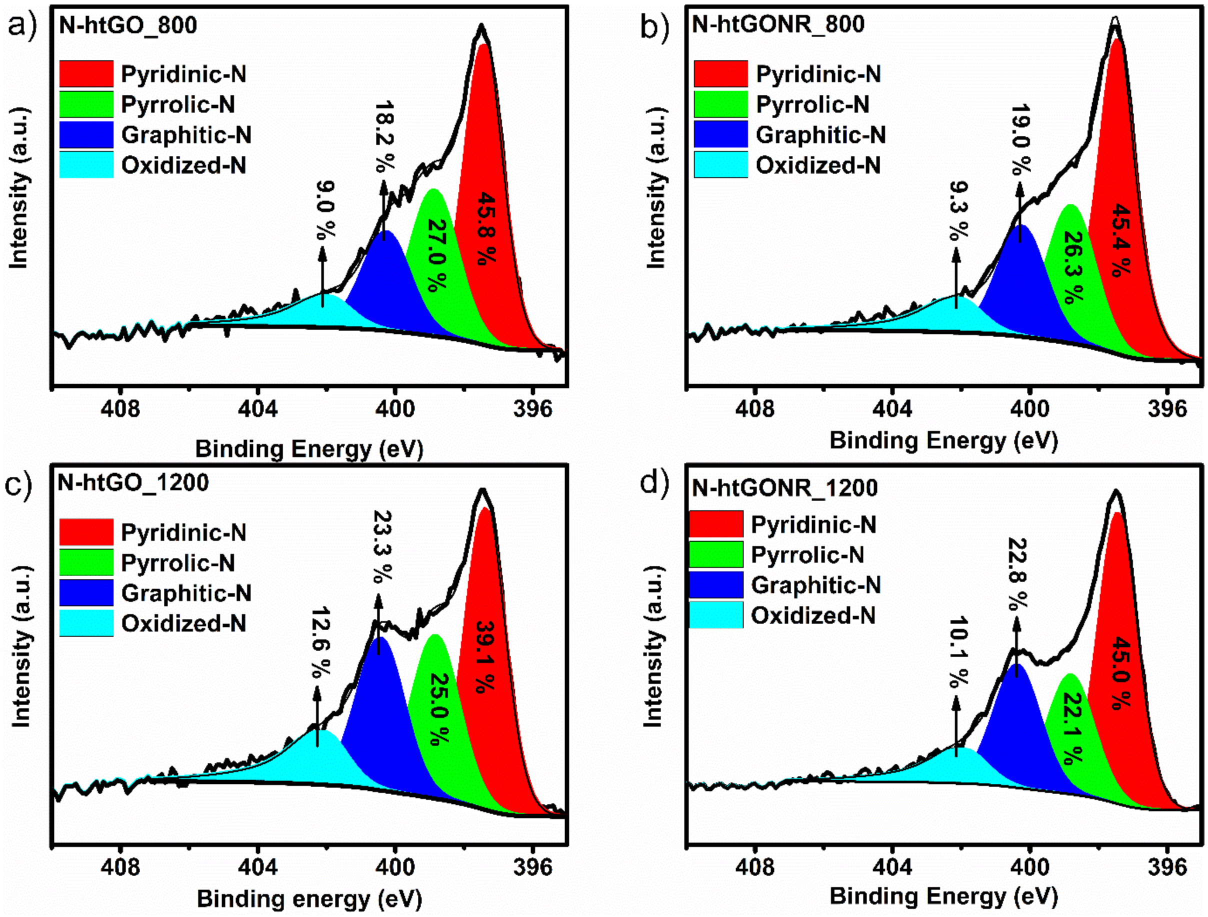

Figure S5: N *1s* fitted XPS spectra of a) N-htGO_800, b) N-htGONR_800, c) N-htGO_1200, and d) N-htGONR_1200 materials. The at% for the N-functionalities are given relative to the total N concentration in the sample.

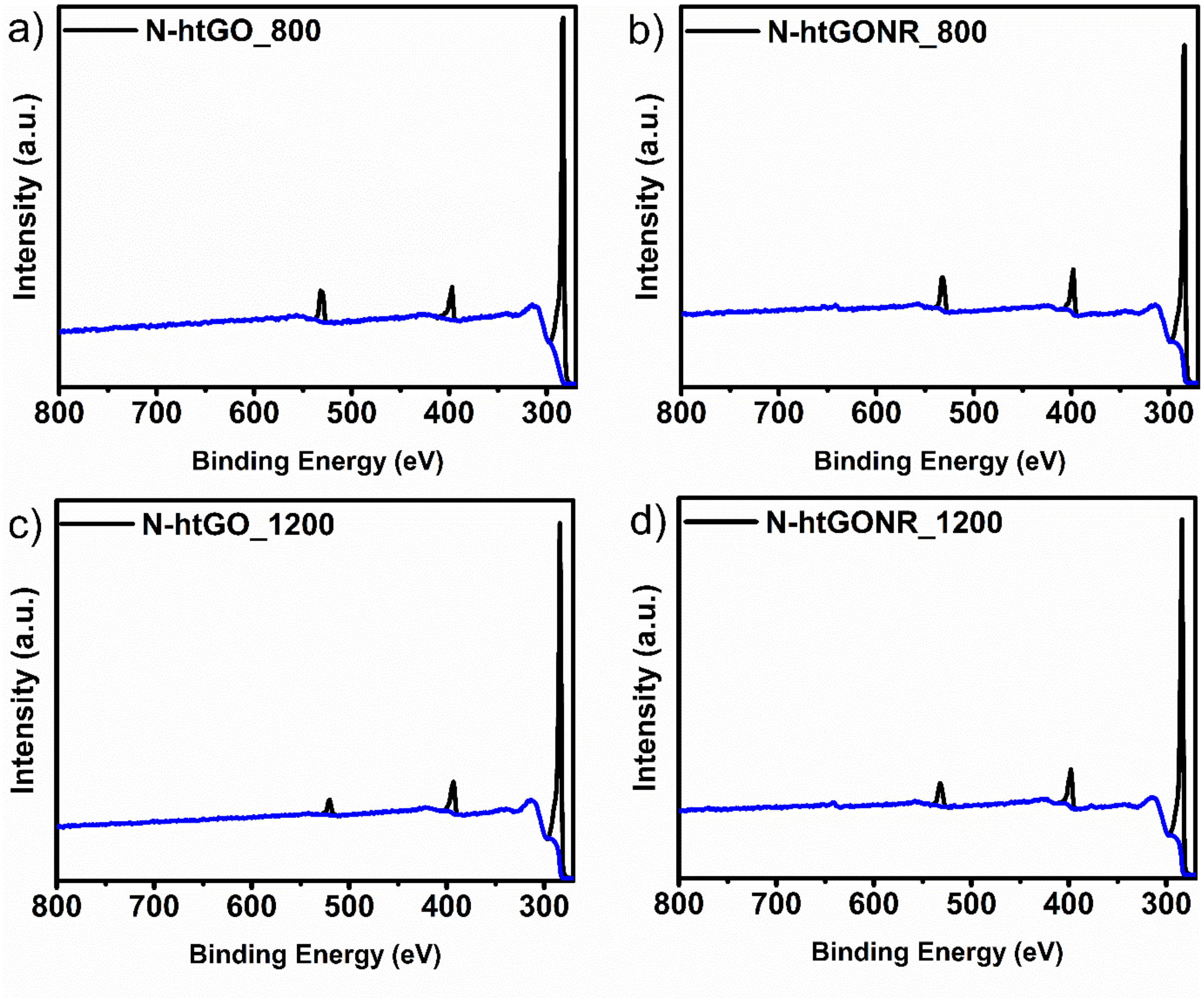

Figure S6: XPS survey spectra of a) N-htGO_800, b) N-htGONR_800, c) N-htGO_1200 and d) N-htGONR_1200 materials.

Table S1: The name and the corresponding Raman peak centre.

Peak Name	Peak Center (cm^{-1})
**D***	1190
**D**	1353
**D****	1500
**G**	1586
**D'**	1610
**G***	2445
**2D**	2690
**D+D***	2925
**2D'**	3160

Table S*2*: Concentrations of trace metal impurities, measured by ICP-MS.

	Elemental concentration (µg/g)						
**Sample**	**Mn**	**K**	**Fe**	**Na**	**P**	**S**	**Pt**
**N-htGO**	767	2089	71	2198	<2	<2	<2
**N-htGONR**	11333	8959	718	<3	<3	<3	<3

Table S3: Atomic surface concentration (at%) of C, O, and N determined by XPS, and E_{onset} in 0.1 M KOH and $HClO_4$ for N-htGO_800, N-htGO_1200, N-htGONR_800, and N-htGONR_1200 samples.

Sample	C (at%)	O (at%)	N (at%)	Pyridinic-N (at%)	E_{onset} (KOH) (V vs. RHE)	E_{onset} ($HClO_4$) (V vs. RHE)
**N-htGO_800**	90.6	3.6	5.8	2.41	0.795	0.324
**N-htGO_1200**	91.7	1.7	6.6	2.27	0.823	0.340
**N-htGONR_800**	89.3	3.8	6.7	3.01	0.841	0.512
**N-htGONR_1200**	91.6	2.6	5.8	3.37	0.863	0.599

Table S4: Conductivity of compressed N-htGO_800, N-htGO_1200, N-htGONR_800 and N-htGONR_1200 films determine by the four-point probe method.

Measurement	σ (S/m) N-htGO_800	σ (S/m) N-htGO_1200	σ (S/m) N-htGONR_800	σ (S/m) N-htGONR_1200
1	26.31	35.71	2.08	7.14
2	27.77	41.67	2.27	7.46
3	25.77	37.04	1.92	7.35
4	25.00	45.46	2.02	7.46
**Average**	**26.18**	**39.60**	**2.07**	**7.35**

Table S5: Comparison of E_{onset} between N-doped graphene derivatives in 0.1 M KOH

Catalyst	E_{onset} (V vs. RHE)	n (0.5 V vs. RHE)	Literature
**Pt/C**	0.98	4	[1]
**N-htGONR_1200**	0.86	3.5	This work
**NGAs**	0.66	3.8	[2]
**rGO-N**	0.74	2.6	[3]
**NG-shNH3**	0.76	3.6	[4]
**NPEGO**	0.81	3.6	[5]
**N-Graphene**	0.53	-	[6]
**NG-900**	0.85	3.6	[7]
**N-graphene_900**	0.86	4.0	[8]
**NbmGO**	0.81	4.0	[9]
**2-NGF-9**	0.90	3.8	[1]

Table S6: Comparison of E_{onset} between N-doped graphene derivatives in 0.1 M $HClO_4$

Catalyst	E_{onset} (V vs. RHE)	n (0.5 V vs. RHE)	Literature
**Pt/C**	1.05	4	[10]
**N-htGONR_1200**	0.60	3.7	This work
**N-Graphene**	0.25	-	[6]
**C-PANI**	0.61	3.9	[10]
**NDC**	0.65*	3.7	[11]
**N-tGO**	0.36	-	[12]

*Measured in 1 M $HClO_4$

Supplementary References

(1) Skorupska, M.; Ilnicka, A.; Lukaszewicz, J. P. The Effect of Nitrogen Species on the Catalytic Properties of N-Doped Graphene. *Sci. Rep.* **2021**, *11* (1), 1–11. https://doi.org/10.1038/s41598-021-03403-8.

(2) Wu, Z. S.; Yang, S.; Sun, Y.; Parvez, K.; Feng, X.; Müllen, K. 3D Nitrogen-Doped Graphene Aerogel-Supported Fe 3O 4 Nanoparticles as Efficient Electrocatalysts for the Oxygen Reduction Reaction. *J. Am. Chem. Soc.* **2012**, *134* (22), 9082–9085. https://doi.org/10.1021/ja3030565.

(3) Zhang, Y.; Fugane, K.; Mori, T.; Niu, L.; Ye, J. Wet Chemical Synthesis of Nitrogen-Doped Graphene towards Oxygen Reduction Electrocatalysts without High-Temperature Pyrolysis. *J. Mater. Chem.* **2012**, *22* (14), 6575–6580. https://doi.org/10.1039/c2jm00044j.

(4) Sasikala, S. P.; Huang, K.; Giroire, B.; Prabhakaran, P.; Henry, L.; Penicaud, A.; Poulin, P.; Aymonier, C. Simultaneous Graphite Exfoliation and N Doping in Supercritical Ammonia. *ACS Appl. Mater. Interfaces* **2016**, *8* (45), 30964–30971. https://doi.org/10.1021/acsami.6b10570.

(5) Wang, Y.; Yu, F.; Zhu, M.; Ma, C.; Zhao, D.; Wang, C.; Zhou, A.; Dai, B.; Ji, J.; Guo, X. N-Doping of Plasma Exfoliated Graphene Oxide: Via Dielectric Barrier Discharge Plasma Treatment for the Oxygen Reduction Reaction. *J. Mater. Chem. A* **2018**, *6* (5), 2011–2017. https://doi.org/10.1039/c7ta08607e.

(6) Bai, J.; Zhu, Q.; Lv, Z.; Dong, H.; Yu, J.; Dong, L. Nitrogen-Doped Graphene as Catalysts and Catalyst Supports for Oxygen Reduction in Both Acidic and Alkaline Solutions. *Int. J. Hydrogen Energy* **2013**, *38* (3), 1413–1418. https://doi.org/10.1016/j.ijhydene.2012.11.039.

(7) Lin, Z.; Song, M. K.; Ding, Y.; Liu, Y.; Liu, M.; Wong, C. P. Facile Preparation of Nitrogen-Doped Graphene as a Metal-Free Catalyst for Oxygen Reduction Reaction. *Phys. Chem. Chem. Phys.* **2012**, *14* (10), 3381–3387. https://doi.org/10.1039/c2cp00032f.

(8) Geng, D.; Chen, Y.; Chen, Y.; Li, Y.; Li, R.; Sun, X.; Ye, S.; Knights, S. High Oxygen-Reduction Activity and Durability of Nitrogen-Doped Graphene. *Energy Environ. Sci.* **2011**, *4* (3), 760–764. https://doi.org/10.1039/c0ee00326c.

(9) Vasiliev, V. P.; Manzhos, R. A.; Kochergin, V. K.; Krivenko, A. G.; Kabachkov, E. N.; Kulikov, A. V.; Shulga, Y. M.; Gutsev, G. L. A Facile Synthesis of Noble-Metal-Free Catalyst Based on Nitrogen Doped Graphene Oxide for Oxygen Reduction Reaction. *Materials (Basel).* **2022**, *15* (3). https://doi.org/10.3390/ma15030821.

(10) Peng, H.; Mo, Z.; Liao, S.; Liang, H.; Yang, L.; Luo, F.; Song, H.; Zhong, Y.; Zhang, B. High Performance Fe- and N- Doped Carbon Catalyst with Graphene Structure for Oxygen Reduction. *Sci. Rep.* **2013**, *3*, 1–7. https://doi.org/10.1038/srep01765.

(11) Choi, C. H.; Park, S. H.; Woo, S. I. Binary and Ternary Doping of Nitrogen, Boron, and Phosphorus into Carbon for Enhancing Electrochemical Oxygen Reduction Activity. *ACS Nano* **2012**, *6* (8), 7084–7091. https://doi.org/10.1021/nn3021234.

(12) Li, Z.; Gao, Q.; Zhang, H.; Tian, W.; Tan, Y.; Qian, W.; Liu, Z. Low Content Pt Nanoparticles Anchored on N-Doped Reduced Graphene Oxide with High and Stable Electrocatalytic Activity for Oxygen Reduction Reaction. *Sci. Rep.* **2017**, *7* (February), 1–9. https://doi.org/10.1038/srep43352.
